# Gene targeting by TALEN-induced homologous recombination in goats directs production of β-lactoglobulin-free, high-human lactoferrin milk

**DOI:** 10.1038/srep10482

**Published:** 2015-05-21

**Authors:** Chenchen Cui, Yujie Song, Jun Liu, Hengtao Ge, Qian Li, Hui Huang, Linyong Hu, Hongmei Zhu, Yaping Jin, Yong Zhang

**Affiliations:** 1 College of Veterinary Medicine, Northwest A&F University, Yangling 712100, Shaanxi, China; 2 Key Laboratory of Animal Biotechnology, Ministry of Agriculture, Northwest A&F University, Yangling 712100, Shaanxi, China

## Abstract

β-Lactoglobulin (BLG) is a major goat’s milk allergen that is absent in human milk. Engineered endonucleases, including transcription activator-like effector nucleases (TALENs) and zinc-finger nucleases, enable targeted genetic modification in livestock. In this study, TALEN-mediated gene knockout followed by gene knock-in were used to generate BLG knockout goats as mammary gland bioreactors for large-scale production of human lactoferrin (hLF). We introduced precise genetic modifications in the goat genome at frequencies of approximately 13.6% and 6.09% for the first and second sequential targeting, respectively, by using targeting vectors that underwent TALEN-induced homologous recombination (HR). Analysis of milk from the cloned goats revealed large-scale hLF expression or/and decreased BLG levels in milk from heterozygous goats as well as the absence of BLG in milk from homozygous goats. Furthermore, the TALEN-mediated targeting events in somatic cells can be transmitted through the germline after SCNT. Our result suggests that gene targeting via TALEN-induced HR may expedite the production of genetically engineered livestock for agriculture and biomedicine.

Goat’s milk has become an alternative to cow’s milk as a source of nutrients for humans, particularly infants, because of its specific composition; however, this milk may also cause an allergic reaction similar to that of cow’s milk. β-Lactoglobulin (BLG) is a major whey protein of the milk from goats and other ruminants, but it is normally absent in human milk. BLG is considered a dominant milk allergen^1^. Despite the use of methods such as heat processing and enzymatic hydrolysis for reducing the allergenic potential of BLG, these biochemical approaches are costly and produce unexpected by-products that may affect milk quality^2^,^3^,^4^. By contrast, genetic modification is a more direct approach to reduce BLG levels in milk.

Conventional gene targeting through homologous recombination (HR) is an effective method for precise genomic modification. HR has been routinely used for the successful and efficient genetic modification of mouse embryonic stem cells to generate gene knockout and knock-in mice^5^,^6^. Few studies have also demonstrated gene targeting in primary somatic cells followed by embryonic cloning to produce genetically modified livestock^7^,^8^,^9^,^10^. Despite the reported viability, this approach exhibits low efficiency, and the technical difficulties involved produce low rates of spontaneous HR. Engineered endonucleases (EENs), including zinc-finger nucleases (ZFNs) and transcription activator-like effector nucleases (TALENs), have emerged as novel and broadly applicable tools for targeted genome editing in living cells or organisms. These endonucleases generate DNA double-strand breaks (DSBs) at preferred genomic regions; DSBs can be repaired by either nonhomologous end joining (NHEJ) or HR. NHEJ often results in gene disruption by introducing small insertions and/or deletions (indels) into the genome. NHEJ-mediated gene disruption has been reported in various species which gene disruption was previously difficult or impossible to perform on^11^,^12^,^13^,^14^,^15^,^16^,^17^, including livestock^18^,^19^,^20^,^21^,^22^,^23^. The bi-allelic modification of *BLG* gene has been achieved in bovine cells by applying ZFNs to introduce NHEJ^21^. Nevertheless, the targeted mutant calves have small in-frame deletions, which do not produce the knockout alleles required for producing BLG-free milk. Alternatively, HR allows for the precise deletion or introduction in the targeted site. Conventional gene targeting approaches have been developed using EENs to perform efficient HR at preferred sites. TALEN-induced HR has been used to introduce a promoter-less expression cassette in pigs for stable gene overexpression^24^. Moreover, we successfully inserted exogenous genes into intron 2 of the beta-casein locus in cows by gene targeting via ZFN-induced HR^25^,^26^. However, to the best of our knowledge, the production of genetically modified livestock using gene knockout followed by gene knock-in has never been reported to date.

Human lactoferrin (hLF) is a multifunctional glycoprotein involved in iron absorption in the intestinal tract as well as in the non-specific immune system^27^,^28^. In the present study, an effective method of using TALEN-mediated gene targeting in goat primary fibroblasts was described. Precise modifications were introduced by TALEN-induced HR to generate cloned goats which expressed decreased BLG levels or/and high levels of hLF. After disruption of one allele, sequential gene targeting was applied to generate BLG knockout goats as well as knock-in goats that secreted hLF at high levels. Furthermore, the genetic modification could be transmitted through the germline.

## Results

### NHEJ-mediated gene disruption in goat fetal fibroblasts

To knockout BLG, we selected exons 1 and 2 of the goat BLG gene as the candidate sequences for the target sites. Two pairs of TALENs were designed and assembled as previously described^29^ (Figure S1). An RFP–GFP reporter system was used to determine the nuclease activities of these EENs in 293FT cells. By contrast, the cells transfected with TALENs and their corresponding reporter constructs yielded appreciable levels of fluorescence, which indicated that these designed nucleases specifically cleaved the target site and induced NHEJ (Figure S2).

To introduce the frame-shift mutations that lead to the production of null alleles via TALENs, two pairs of TALENs were delivered into goat fetal fibroblasts (GFFs) by electroporation to introduce NHEJ as previously described in pigs and calves^18^,^21^. Limited dilution was also used to form cloned cells. Meanwhile, three pairs of ZFNs against the goat *BLG* gene were introduced into GFFs; the activities of the ZFNs were measured in a budding yeast-based system (Sigma-Aldrich) and in 293FT cells (Figures S1 and S2). No mutants were detected when a ~300 bp genomic fragment was amplified and sequenced from the clones in any groups (data not shown). Moreover, GFFs were averse to dilution cloning and most of the derived clones exhibited symptoms of aging.

### HR-mediated gene targeting by using TALENs in GFFs

TALENs can significantly stimulate HR in the primary somatic cells of various livestock^24^,^30^. Therefore, we speculated that TALENs combined with conventional gene targeting may be useful for genetic modification through HR in primary GFFs.

**Figure 1:**
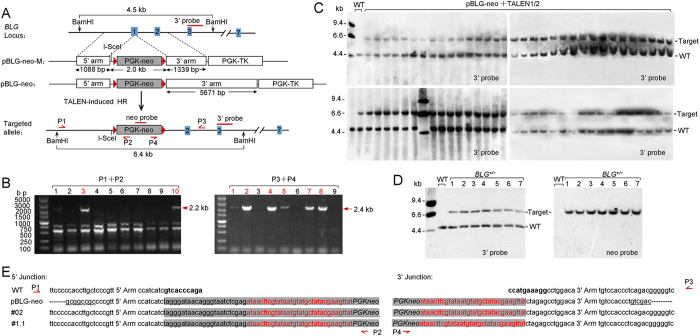
Gene targeting of the *BLG* locus by TALENs in goat fetal fibroblasts. (**A**) Schematic overview showing the targeting strategy. Blue boxes, exons of the *BLG* gene; red boxes, probes used for Southern blot analysis; red triangles, *loxP* sites. (top) Schematic of the *BLG* locus and the targeting vectors with different homologous arms. (bottom) Schematic of the targeted integration of the PGK-neo cassette. For 5′ junction PCR analysis, primers P1 and P2 were located upstream of the 5′ arm and in the *neo* gene, respectively; for 3′ junction PCR analysis, primers P3 and P4 were located at the 3′ arm of the *neo* gene and downstream of the 3′ arm, respectively. The primer positions are indicated on the schematic of the targeted *BLG* locus. For Southern blot analysis, genomic DNA was digested with *Bam*HI; the corresponding expected band sizes are indicated. (**B**) Junction PCR analysis for screening the targeting events with the donor plasmid pBLG-neo-M. Correctly targeted clones are colored red. (Left) 5′ junction PCR analysis of drug-resistant clones. A representative panel of 10 clones had the expected 2.2 kb band. (Right) Genomic DNA of the 5′ junction PCR-positive clones was subjected to 3′ junction PCR. A representative panel of 9 clones had the expected 2.4 kb band. (**C**) Southern blot analysis of junction PCR-positive clones. Wild-type (WT) goats have a unique 4.5 kb band. Junction PCR-positive clones had an additional 6.4 kb band for the correctly targeted allele. (**D**) Southern blot analysis of *BLG*^*+/−*^ goats. The neo probe detected a unique 6.4 kb band for the PGK-neo cassette targeted integration. (**E**) Sequence comparison at the 5′ and 3′ junctions among the wild-type genomic DNA and the donor DNA sequences of a representative *BLG*^*+/−*^ goat (#02) and a representative F1 (#2.1: offspring of founder #02). Restriction sites in 5′ and 3′ ends of the donor DNA are underlined). The exogenous cassettes are colored gray, and the *loxP* sites are colored red. Letters in boldface indicate the 20 bp deletion of the *BLG* gene.

To disrupt the goat *BLG* gene via TALEN-induced HR, the gene-targeting vectors were designed based on the target site of TALEN1/2 (Fig. 1A). The knockout vector pBLG-neo-M contained a neomycin resistance (*neo*) gene as a positive selection marker and a herpes simplex virus type 1 thymidine kinase (*tk*) gene as a negative selection marker. The *neo* gene was flanked by two homologous arms, 1.1 kb for the 5′ arm and 1.3 kb for the 3′ arm. The *loxP* sites were arranged to flank the *neo* gene, which resulted in the removal of the gene after Cre-mediated recombination. In conventional gene targeting, the targeting efficiency can be significantly improved by increasing the length of homologous arms. A longer arm of homology is more effective for searching the target locus and for spontaneous homologous recombination^31^. Consequently, we replaced the 3′ arm of pBLG-neo-M with a 5.3 kb homologous sequence to construct the targeting vector pBLG-neo (Fig. 1A). In the targeted cells, the 20 bp fragment near the signal peptide sequence of the *BLG* gene was deleted and replaced with the PGK-neo cassette, which resulted in a loss-of-function mutation. Polymerase chain reaction (PCR) was used to screen drug-resistant cells for targeting events. The positions of the PCR primers are indicated in Fig. 1. Efficiently amplifying longer targets (>5 kb) with Taq DNA polymerase is difficult; thus, only the 5′ junction PCR with primers P1/P2 was used to screen targeting events with the vector pBLG-neo. The sizes of the expected PCR product in correctly targeted cells were 2.2 and 2.4 kb for the 5′ and 3′ junction PCRs, respectively. We also designed hybridization probes to confirm the goat BLG gene targeting via Southern blot analysis (Fig. 1A).

To test whether the *BLG* gene could be disrupted by spontaneous HR, we delivered the targeting vector pBLG-neo into GFFs via electroporation. We selected 300 drug-resistant clones, but none of these clones were correctly targeted. TALENs were then introduced into GFFs with the targeting vectors to improve targeting efficiency. As summarized in Table 1, by using 5′ junction PCR analysis of drug-resistant clones, the HR efficiency with pBLG-neo was 18.3% (*n* = 526) as compared with the pBLG-neo-M at 16.0% (*n* = 589). Genomic DNA from the 5′ junction PCR-positive clones of the pBLG-neo-M groups was subjected to 3′ junction PCR analysis to exclude false positives, and 13.6% (*n* = 589) of the clones passed the analysis (Fig. 1B). Several clones were inappropriate for subsequent assays because of cell senescence during expansion. We selected the clones that passed the two rounds of PCR for Southern blot analysis to confirm that these clones were integrated into the transgene cassette in the heterozygous form at the *BLG* loci (Fig. 1C). The results suggested that the 5′ and 3′ junction PCR analyses were reliable for screening gene targeting events.

**Figure 2:**
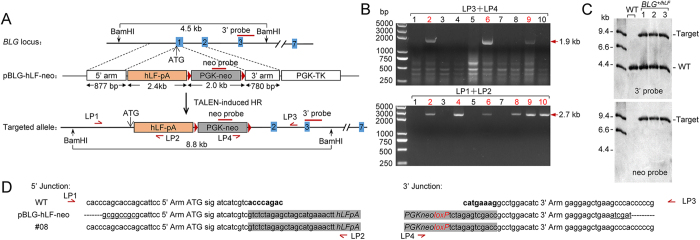
Targeted replacement of the *hLF* gene with the *BLG* locus in goat fetal fibroblasts. (**A**) Schematic overview of the knock-in strategy for the *BLG* locus. (top) Schematic representation of the *BLG* locus and the targeting vector with the hLF cDNA and bovine growth hormone polyadenylation signals. (bottom) Schematic of the targeted integration of *hLF* gene and PGK-neo cassette. (**B**) Junction PCR analysis for screening the targeting events by using donor pBLG-hLF-neo. The positions of primers for the junction PCRs are indicated on the knock-in locus schematic. Correctly targeted clones are in red. (top) Analysis of drug-resistant clones by 3′ junction PCR with primers LP3 and LP4. A representative panel of 10 clones showed the expected 1.9 kb band. (bottom) Genomic DNA of the 3′ junction PCR-positive clones was PCR-amplified with primers LP1 and LP2. A representative panel of 10 clones showed the expected 2.7 kb band. (**C**) Southern blot analysis of *BLG*^*+/−*^ goats. Genomic DNA was digested with *Bam*HI and hybridized with the external 3′ probe or the internal neo probe. The 3′ probe detects a 4.5 kb band for the wild-type and an 8.8 kb band for the *hLF* targeted integration. The neo probe detects the unique 8.8 kb band for the targeted integration. (**D**) Sequence comparison of the 5′ and 3′ junctions between the wild-type genomic DNA and the donor DNA of a representative *BLG*^*+/hLF*^ goat (#L2). Restriction sites in the 5′ and 3′ ends of the donor DNA are underlined. The 5′ and 3′ ends of the expression cassette are colored gray; the *loxP* sites are identical to those in Fig. 1E. Letters in boldface indicate the 16 bases of the *BLG* gene that were replaced by the hLF gene and the PGK-neo cassette. “sig” indicates the signal peptide sequence of the *BLG* gene.

The *BLG* gene is supposed to be an ideal locus for mammary gland bioreactors. We investigated whether the exogenous gene can be expressed under the control of the endogenous *BLG* promoter. To introduce the human lactoferrin (hLF) gene into the BLG locus, the targeting vector pBLG-hLF-neo was constructed by inserting the hLF cDNA with bovine growth hormone polyadenylation signals between the 5′ arm and the selection marker (Fig. 2A). Co-electroporation of the vector with TALEN1/2 into GFFs resulted in correctly targeted clones with an efficiency of 12.5% (*n* = 799; Table 1), as determined by junction PCR analysis (Fig. 2B).

### Generating mono-allelic targeted goats

To eliminate the possible genomic integration of TALEN–DNA constructs, we applied TALEN-encoding mRNAs to generate targeted cells to be used as donor nuclei for SCNT. In total, PCR analysis showed that 9.90% (*n* = 980) of all the drug-resistant clones harbored the correctly targeted allele (Table S1). The *in vitro* developmental experiment revealed differences in the blastocyst formation among the groups (Table S2). We chose the clones such as 0622E4, which had a significantly higher blastocyst rate to produce cloned goats (Table S2). The karyotype of each clone was checked, and all types exhibited normal chromosome numbers. A total of 10 cloned offspring were obtained from 70 recipients; these offspring presented birth weights ranging from 2.4 kg to 4.1 kg (Table S3) and all of them have grown to adulthood. Genomic DNA analysis of these cloned goats via PCRs and Southern blots confirmed the successful targeting of seven *BLG*^*+/−*^ and three *BLG*^*+/hLF*^; the exogenous DNA was integrated in the heterozygous form at the *BLG* locus without random integration of the targeting vectors in cloned goats (Fig. 1D and 2C). By sequencing the products of 5′ and 3′ junction PCR, we found that a 20 bp deletion of the *BLG* gene and a 1,935 bp selection gene were introduced into the cognate chromosomal location in *BLG*^*+/−*^ goats (Fig. 1E); the 2,379 bp *hLF* gene was inserted after the signal peptide sequence of the *BLG* gene in *BLG*^*+/hLF*^ goats (Fig. 2D) and replaced 16 bases of the *BLG* gene. To investigate whether the untargeted allele was modified by NHEJ, we examined all cloned offspring via PCR-based DNA sequencing. The results did not identify NHEJ-induced mutations in the intact allele.

### **Targeting the second allele of** ***BLG***

**Figure 3:**
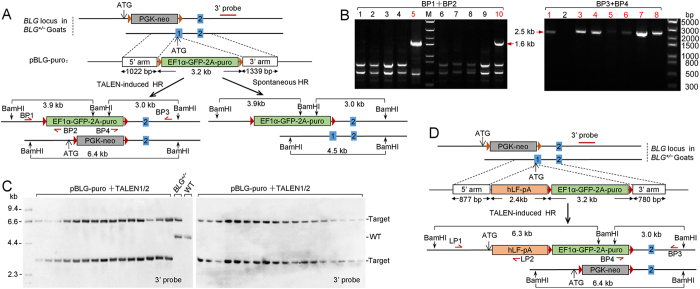
Targeting the second allele of the *BLG* gene in *BLG*^*+/−*^ fibroblasts. (**A**) Schematic overview of the bi-allelic knockout strategy for generating null *BLG* alleles. (top) Schematic of the targeted *BLG* locus in *BLG*^*+/−*^ goats and the secondary knockout vector with the puromycin selection marker. (bottom) Schematic of the exogenous cassette integration. (left) Targeted alleles are generated by pBLG-puro via TALEN-induced homologous recombination with the intact *BLG* allele. (right) Targeted alleles are generated by pBLG-puro via spontaneous homologous recombination with the PGK-neo integrated allele. (**B**) Junction PCR analysis for screening the gene targeting events with the donor pBLG-puro plasmid. (**C**) Southern blot analysis of clones via positive junction PCR with the knockout vector pBLG-puro to analyze homologous recombination events. Genomic DNA was digested with *Bam*HI and hybridized with the external 3′probe. Positive clones show a 6.4 kb band for the PGK-neo cassette integration allele and a 3.0 kb band for the EF1α-GFP-2A-puro cassette integration allele. (**D**) Schematic of the targeting strategy for knock-in modification following gene knockout. (top) Schematic of the targeted *BLG* locus in *BLG*^*+/−*^ goats and the secondary knock-in vector with the puromycin selection marker. (bottom) Schematic of the targeted alleles.

To target the second allele of the *BLG* gene, we prepared a secondary targeting vector containing the puromycin (puro) selection marker for generating null *BLG* allele (Fig. 3A). *BLG*^*+/−*^ fibroblasts were isolated from the ear tissues of mono-allelic targeted goats. TALEN–DNA constructs were introduced to form DSBs at the intact allele in *BLG*^*+/−*^ cells which produced bi-allelic targeting events via the secondary vector. Junction PCRs analysis with the primers BP1/BP2 and BP3/BP4 of puro-resistant clones revealed that 6.09% (*n* = 673) of these clones were correctly targeted (Table 1 and Fig. 3B). During targeting of the second allele, the secondary vector possibly underwent spontaneous HR with the integrated targeting vector and replaced the PGK-neo cassette in the previously targeted allele rather than disrupting the intact allele (Fig. 3A). Consequently, the genomic DNA from PCR-positive clones was subjected to Southern blot analysis to verify this possibility. As shown in Fig. 3C, all clones exhibited two integrated alleles with the 6.4 and 2.9 kb bands that correspond to the integration alleles; thus, the secondary targeting vector could undergo TALEN-induced HR only with the intact allele (Fig. 3B). In contrast to the first gene targeting in GFFs, we observed that negative drug selection could not enrich the targeting events but could facilitate the senescence of cell clones (Table 1).

**Figure 4:**
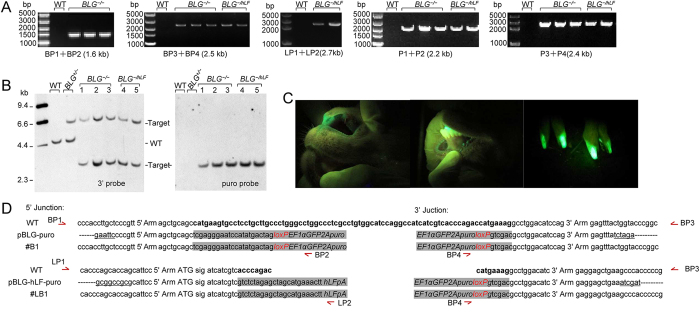
Identification of the bi-allelic targeted goats. (**A**) Junction PCR analysis of bi-allelic targeted goats. (**B**) Southern blot analysis of bi-allelic *BLG*-targeted goats. Genomic DNA was digested with *Bam*HI and hybridized with the external 3′probe or a puro probe. The puro probe detects a unique 3.0 kb band after targeted integration of the EF1α-GFP-2A-puro cassette. (**C**) GFP expression in the *BLG*^*−/−*^ goat (#B1) was observed using a Dual Fluorescent Protein Flashlight (NightSea DFP-1, MA, USA). (**D**) Sequence comparison at the 5′ and 3′ junctions with wild-type genomic DNA and donor DNA sequences of a representative *BLG*^*−/−*^ goat (#B1) and a *BLG*^*−/hLF*^ (#LB1), respectively. Restriction sites in the 5′ and 3′ ends of the donor DNA are underlined. The 5′ and 3′ ends of the expression cassette are colored gray; the *loxP* sites are identical to those in Fig. 1E. Letters in boldface indicate the 80 deleted bases of the *BLG* gene and the 16 replaced bases of the *BLG* gene after the second gene targeting.

To replace the intact *BLG* allele with the *hLF* gene in *BLG*^*+/−*^cells, we constructed another secondary targeting vector, pBLG-hLF-puro (Fig. 3D). TALEN-encoding mRNAs and the secondary vectors were used to prepare the bi-allelic targeted cells as donor cells for SCNT (Table S1). Five cloned goats were produced and survived for more than three months (Table S3). Junction PCR analysis revealed that three goats were *BLG*^*−/−*^ and the remaining goats were *BLG*^*−/hLF*^ (Fig. 4A). Southern blot analysis of the genomic DNA from these cloned goats suggested that they harbored the two targeted alleles (Fig. 4B). Fig. 4C illustrates the level of GFP expression in the skin of bi-allelic targeted goats. DNA sequencing indicated that the 80-bp fragment containing the ATG start codon of the goat *BLG* gene was replaced with the 3,153 bp exogenous fragment; the *hLF* gene was inserted in the same site in the *BLG*^*+/hLF*^ goats (Fig. 4D).

### **Analysis of milk from** ***BLG*** **-targeted goats**

**Figure 5:**
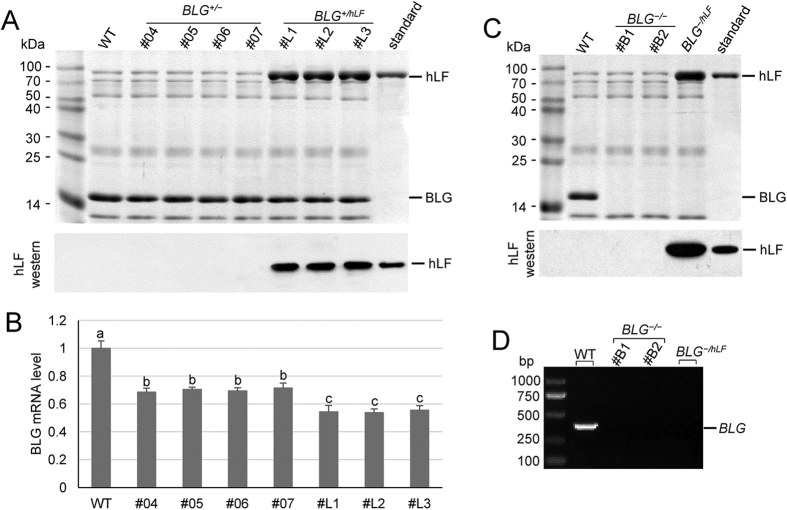
Effects of BLG knockout in goat’s milk. (**A**) Analysis of milk from mono-allelic *BLG*-targeted goats. (top) Analysis of whey proteins by Coomassie blue staining after separation by SDS/PAGE. Equal amounts of milk samples were loaded onto each lane of the gel. (bottom) Western blot analysis of hLF expression. (**B**) Analysis of BLG expression through qPCR. Values with different superscripts are significantly different (*P* < 0.05). Three assay replicates were performed for each milk sample. (**C**) Analysis of milk from bi-allelic *BLG*-targeted goats. (top) Analysis of whey proteins by Coomassie blue staining after separation by SDS/PAGE. Equal amounts of milk samples were loaded onto each lane of the gel. (bottom) Western blot analysis of hLF expression. (**D**) Reverse-transcription PCR analysis of BLG mRNAs in cells from the milk of bi-allelic *BLG*-targeted goats.

To evaluate BLG expression and hLF expression in mono-allelic targeted goats, we bred *BLG*^*+/−*^ goats to induce natural lactation (Table 2) and *BLG*^*+/hLF*^ goats were hormonally induced into lactation. Casein proteins were removed from the defatted milk samples via acid precipitation, and whey proteins were analyzed by SDS gel electrophoresis and Coomassie blue staining. The cells in milk were collected to determine the BLG mRNA levels. As shown in Fig. 5A, BLG was immediately detected as a major whey protein in the wild-type control. By contrast, BLG expression in milk from mono-allelic targeted goats was reduced. Quantitative real-time PCR (qPCR) revealed that BLG expression was s downregulated by 28%–31% in *BLG*^*+/−*^ goats and by 43%–45% in *BLG*^*+/hLF*^ goats (Fig. 5B). Compared with normal goat’s milk, an additional~80 kDa band was present in milk samples from *BLG*^*+/hLF*^ goats; revealed that the extra protein was hLF (Fig. 5A). The hLF concentrations were ~2.3, 2.3, and 2.4 mg/mL in the milk of goats #L1, #L2, and #L3, respectively, as measured by enzyme-linked immunosorbent assay (ELISA).

To confirm the knockout of BLG expression, we hormonally induced bi-allelic targeted goats into lactation. Unlike the normal milk samples, BLG was absent in the milk from *BLG*^*−/−*^ and *BLG*^*−/hLF*^ goats, whereas hLF was observed in milk from *BLG*^*−/hLF*^ goats (Fig. 5C). Consistent with the results obtained via SDS-PAGE, BLG mRNAs were not detected via reverse-transcription PCR in all the bi-allelic targeted goats (Fig. 5D). As detected by ELISA, the hLF expression levels in milk from *BLG*^*−/hLF*^ goats were higher than in milk from *BLG*^*+/hLF*^ goats (~3.2 mg/mL).

### **Germline transmission of the** **targeting events**

We also investigated whether the targeted allele of the *BLG* gene can be transmitted through the germline. At ~15 months of age, the naturally mature male *BLG*^*+/−*^ founder #02 was crossed with four wild-type goats and four *BLG*^*+/−*^ founders. Nine F_1_ offspring were born, and their genotypes were monitored via PCR. As expected, all progenies were wild type or inherited one or two mutant alleles from their parents (Table 2).

## Discussion

BLG is considered a major milk allergen, but its nutritional and biological functions remain unknown. The production of BLG knockout dairy livestock will enable the direct evaluation of the allergenicity of BLG as well as the full investigation of its biological function. Despite its being an attractive target gene, BLG knockout animals have not been generated to date. This report demonstrated the efficient modification of the *BLG* gene via gene targeting with TALEN-induced HR in goat primary cells. For the first time, we successfully knocked out BLG in goats and confirmed the germline transmission of the targeting events through HR.

EENs disrupt various endogenous genes in primary cells of livestock by introducing NHEJ^18^,^19^,^20^. The successful bi-allelic modification of the *BLG* gene in bovine cells was previously conducted without antibiotic selection^21^. However, we failed to isolate the genome-modified cells with ZFNs or TALENs in the present study, although their activities were confirmed. In a previous work, NHEJ-induced indels declined by 50% to 90% after the culture was extended without antibiotic selection in bovine cells^20^. In addition, goat primary cells are averse to dilution cloning and low culture density. Thus, we speculated that the EEN activity was not the cause of the failed modification of the *BLG* gene in GFFs. The co-transfection of cells with EENs and assistant vectors with an antibiotic resistant marker efficiently enriched modified cells in previous studies^19^,^20^,^32^. However, the random integration of the assistant vector could not be prevented.

Despite the advantages of NHEJ-induced modification, indels are unpredictable and variable; thus, precise genomic alterations are not allowed, and screening for functional loss mutants becomes arduous, particularly for transcriptionally active genes. Alternatively, gene targeting through highly reliable HR can introduce precise deletions and insertions into the genome when a homologous DNA plasmid template is used. Since the first study on gene targeting in sheep in 2000^7^, limited success has been demonstrated in other livestock species^8^,^9^,^10^. A previous report stated that the frequency of a conventional method to target a silent gene in cattle fibroblasts is only 0.45% when vectors with approximately 7.2 kb homology are used^10^. In the present study, the use of targeting vectors with short homologous sequences (a total of ~2 kb) achieve targeting events at a frequency of 13% with TALEN-induced HR. Importantly, the short arms facilitated the screening process and guaranteed the reliability of the PCR-based analysis. TALEN-induced HR can be affected by the distance between the homology arms and the DNA cleavage site. Microinjection of TALENs and ssDNA oligonucleotides (ssODNs) into one-cell mouse embryos revealed that nucleotide replacements preferentially occur in proximity to the DSB site but are found within a distance of up to 44 bp^33^. This replacement may hinder the exchange between the endogenous sequences and the donor template because ssODNs can function as effective templates for HR but are generally 40–200 nt in length. A previous study that used a DNA vector as a template showed that the distance does not affect the targeting efficiency in rat zygotes^34^. Consistent with this finding, the present study showed that the efficiency of HR using homologous arms contiguous to the DSB site (~17 bp) was similar to the efficiency when the homology was ~80 bp from the DSB site *in vitro*. These results suggested that the precise HR-mediated deletion or insertion with DNA templates is not limited to DSBs and may have more extensive applications.

Exogenous DNA can be precisely inserted into a specific site of the genome by TALEN-mediated gene targeting, but the integration of selectable marker genes may cause several problems, such as increasing the public concern regarding the release of antibiotic-resistance genes into the environment and confounding the food safety evaluation of these transgenic animals. In the present study, we introduced the *loxP* sites to flank the selectable marker genes, which allowed for the removal of the marker genes after Cre-mediated recombination. Consequently, we successfully produced antibiotic-selectable marker-free transgenic cattle by using the cell-permeant Cre protein^35^.

Based on the advance of cloning and transgenic techniques, various recombinant proteins have been produced via the mammary gland bioreactors of goats, cattle, pigs or rabbits^36^. Exogenous genes are routinely fused to the regulatory elements of mammary gland-specific genes before they are randomly transferred into the animals. The expression levels of the same foreign protein usually vary among transgenic animals that have been generated by the same method^37^,^38^. Transgene expression can be influenced by the local environment (position effects), which can lead to silencing or aberrant expression of transgenes^39^,^40^,^41^,^42^. For large-scale production, cattle are the most ideal animals as mammary bioreactors. However, the long gestation period of cows leads to an extensive waiting period before the transgenic lines are established, and animal production is very expensive. Consequently, goats are attractive alternative bioreactors. In the present study, the mammary glands of precisely genetically modified goats secreted hLF at a high and stable level; multiple site-specific genetic modification was accomplished within a shorter time period by sequential gene targeting as compared with traditional breeding strategies. After one copy of the *BLG* allele was disrupted, BLG protein expression was reduced by an average of 30%, rather than the expected 50%, in *BLG*^*+/−*^ goats. Interestingly, the *BLG* gene replacement with hLF cDNA induced large-scale hLF expression and almost 45% reduction of BLG expression in the milk from *BLG*^*+/hLF*^ goats. The hLF expression in *BLG*^*−/hLF*^ goats was increased because both *BLG* alleles were disrupted. We speculate that the expression of the intact *BLG* allele was upregulated to compensate for the inactive allele in mono-allelic targeted goats; the increased hLF expression also compensated to balance the milk protein synthesis.

The germline transmission of transgenic mutations is an important concern. The TALEN-induced direct modification of zygotic genomes can be transmitted through the germline to the next generation^43^. Although various TALEN-induced genetically modified livestock were previously established through somatic cell modification and cloning, the transmission of TALEN-mediated targeting events in somatic cells through the germline after nuclear transfer remained ambiguous. In the present work, the modifications were stably transmitted to the progeny by mating *BLG*^*+/−*^ goats with the wild-type or *BLG*^*+/−*^ goats. This finding is important for animal breeding or the production of mammary bioreactors.

In summary, our results demonstrated that NHEJ-induced mutations are inefficient in primary cells that resist dilution cloning. Gene targeting through TALEN-induced HR is an efficient approach to introduce precise genetic modification, which knocks out protein production in the mammary glands of livestock. Compared with breeding to produce homozygous animals, sequential gene targeting can provide multiple genetic modifications for different purposes within a reduced time period. For the first time, we applied gene knockout followed by gene knock-in to generate BLG-free goats as mammary gland bioreactors for the large-scale production of hLF. We anticipate that this germline-transmittable genetic modification in livestock will provide a powerful platform for agricultural or biomedical applications.

## Methods

### Ethics Statement

All experiments were approved by the Care and Use of Animals Center of the Northwest A&F University. This study was carried out in strict accordance with the Guidelines for the Care and Use of Animals of Northwest A&F University. The goat ovaries were collected from the Tumen abattoir, a local slaughterhouse of Xi’An, P. R. China. The fetuses and recipient goats were obtained from Yangling Keyuan Cloning Co., Ltd. Every effort was made to minimize animal pain, suffering, and distress as well as to reduce the number of animals used. All surgery was performed under anesthesia by intravenous injection of sumianxin, a compound anesthetic containing dimethylaniline thiazole, dihydroetorphine hydrochloride, ethylenediaminetetraacetic acid, and haloperidol (0.01 mL/kg; Veterinary Research Institute, Jilin, China).

### Design and production of TALENs

Customized TALENs against the β-lactoglobulin gene (BLG, GenBank: Z33881) were designed. The candidate target site was selected by the “TAL Effector-Nucleotide Targeter”^44^. DNA constructs of TALENs were produced by the “unit assembly” method^29^. Transfection grade plasmids were prepared with the Promega PureYield Plasmid Midiprep System (Promega). The assembled TALEN vectors were linearized by *Not*I to be used as templates for the *in vitro* TALEN mRNA transcription with the AmpliCap™ SP6 High Yield Message Maker Kit (Epicentre). The synthesized mRNA was purified with the RNeasy Mini Kit (Qiagen).

### Targeting vector construction

To disrupt the *BLG* locus, the knockout vectors pBLG-neo and pBLG-neo-M contained a neomycin resistance gene (*neo*) driven by a phosphoglycerol kinase (PGK) promoter flanked by two homologous arms contiguous to the TALEN cleavage point. By contrast, the knockout vector pBLG-puro contained a EF1α-GFP-2A-puromycin cassette flanked by two homologous arms at ~80 bp from the TALEN cleavage point. To replace the *BLG* gene, the hLF cDNA-bGH pA was inserted between the 5′ arm and the selection marker gene.

### Cell transfection and selection

GFFs were isolated from 35–40-day-old fetuses and maintained in DMEM/F12 supplemented with 10% FBS at 37 °C in a 5% CO_2_ environment. At 70%–80% confluence, passage 3 cells were harvested by trypsinization. Each transfection reaction contained 1 × 10^6^ cells resuspended in 200 μL electroporation working buffer (electroporation buffer:opti-MEM (Gibco) = 3:1, v/v; electroporation buffer: 120 mM of KCl, 0.15 mM of CaCl_2_, 10 mM of K_2_HPO_4_, and 5 mM of MgCl_2_) with 5 μg of the respective TALEN plasmid DNA or mRNA and 10 μg of the donor plasmid DNA; transfection was facilitated by electroporation with a BTX ECM2001 apparatus (input voltage: 500 V; pulse width: 1 ms; pulse number: 1). The electroporated cells were plated onto 10 cm plates with 1 × 10^5^ cells per plate. The selection medium (700 μg/mL of G418 or 0.8 μg/mL of puromycin) was applied at 48 h after plating and replaced every 3–4 days.

### Detection of gene targeting events by PCR analysis

Drug-resistant cell clones derived from the transfected cell populations were collected by trypsinization, and 70% of which were plated in serum-containing culture medium and expanded. The remaining clones were resuspended in 20 μL of PCR-compatible lysis buffer (10 mM of Tris-HCl, pH 8.5; 50 mM of KCl; 1.5 mM of MgCl_2_; 0.5% NP-40; 0.5% Tween-20; 400 g/mL of proteinase K) for PCR analysis. The lysates were incubated at 65 °C for 60 min and then at 95 °C for 15 min.

To distinguish the *BLG*-targeted cell clones, 2 μL of the DNA lysate was added to a PCR reaction with PCR primers for 5′ (or 3′) junction PCR and subjected to PCR with La *Taq* (TaKaRa) for 30 cycles (95 °C, 30 s; 60 °C, 30 s; 72 °C, 90 s). Subsequently, 3′ (or 5′) junction PCR was performed on the 5′ (or 3′) positive clones to confirm the correct targeting events. Clones that passed both tests were expanded, and their genomic DNA was extracted with TIANamp Genomic DNA Kit (Tiangen Biotech) to confirm the targeting events by PCR analysis as described above. The PCR products were cloned into pMD19-T plasmids (TaKaRa) for sequencing (GenScript Co., Ltd.).

### Somatic cell nuclear transfer

Somatic cell nuclear transfer was performed as previously described by our laboratory^45^. Briefly, oocytes with the first polar body were selected for enucleation at 22–24 h after maturation *in vitro*. Both the polar body and metaphase plate were removed, and a single round donor cell was injected into the perivitelline space of the enucleated oocyte. Karyoplast–cytoplast couplets were fused by electrofusion. The couplets were incubated for 2–3 h in TCM-199 supplemented with 10% FBS and 7.5 μg/mL of cytochalasin B. The fused embryos were activated by treatment with 5 μM of ionomycin for 5 min and 2 mM of 6-dimethylaminopurine for 4 h. The activated embryos were extensively washed before they were cultured in 200 μL of mSOF supplemented with 10% FBS while covered with mineral oil at 38.5 °C in a humidified atmosphere with 5% CO_2_. The embryos were cultured for 7 days to evaluate the *in vitro* developmental rate. After cell culture for 20–24 h, selected one- or two-cell stage embryos were transferred into the oviducts of synchronized recipients on day 1 of estrus (day 0 = estrus; 19–28 embryos per recipient). Pregnancy was determined by ultrasonography.

### Induction of Lactation and Analysis of Goat’s Milk

Lactation was hormonally induced in 7-month-old goats according to the previously described protocol^46^. Briefly, animals were given estradiol (0.25 mg/kg, IM) and progesterone (0.75 mg/kg, IM) on days 1, 3, 5, 7, 9, 11, and 13; prednisilone (0.4 mg/kg, IM) was administered on days 14–16 with daily mammary massage from day 5 onwards. After sample collection, hormonally induced lactation was stopped. Milk samples were centrifuged at 3,000 g for 15 min to remove the fat fractions. The supernatant liquids were then adjusted to pH 3.8–4.6 with 1 M HCl to eliminate the casein fraction; the remaining cell pellet was re-suspended in buffer and again centrifuged for 5 minutes at 3000 rpm. The above process was repeated three times. The cell pellet was added into Trizol to extract total mRNAs.

## Additional Information

**How to cite this article**: Cui, C. *et al.* Gene targeting by TALEN-induced homologous recombination in goats directs production of ß-lactoglobulin-free, high-human lactoferrin milk. *Sci. Rep.* **5**, 10482; doi: 10.1038/srep10482 (2015).

## Supplementary Material

Supplementary Information

## Figures and Tables

**Table 1:** Gene targeting by TALENs in goat somatic cells.

^**Cells (sex)**^	^**Nuclease Pairs**^	^**Targeting vector**^	^**Drug-resistant Clones**^	^**5**^′^**junction PCR+ clones (%)**^	^**3**^′^junction PCR+ clones^*^(%)^	^Senescent^^*^
^GFF4 (F)^	–	^pBLG-neo^	^264^	^0^	–	–
	^TALEN1/2^	^pBLG-neo^	^271^	^50 (18.5)^	–	^10^
	^TALEN1/2^	^pBLG-neo-M^	^285^	^45 (15.8)^	^39 (13.7)^	^8^
	^TALEN1/2^	^pBLG-hLF-neo^	^357^	^54(15.1)^	^45 (12.6)^	^13^
^GFF2 (M)^	–	^pBLG-neo^	^243^	^0^	–	–
	^TALEN1/2^	^pBLG-neo^	^255^	^46 (18.0)^	–	^8^
	^TALEN1/2^	^pBLG-neo-M^	^304^	^49 (16.1)^	^41 (13.5)^	^7^
	^TALEN1/2^	^pBLG-hLF-neo^	^422^	^63 (12.9)^	^55 (13.0)^	^15^
^GFF2 (M)^	–	^pBLG-puro^	^115^	^0^	^0^	–
	^TALEN1/2^	^pBLG-puro^	^271^	^42 (15.5)^	^36 (13.3)^	^4^
^GAF1 (M)^	–	^pBLG-puro^	^126^	^0^	^0^	–
	^TALEN1/2^	^pBLG-puro^	^186^	^29 (15.6)^	^25 (13.4)^	^4^
^TGAF1 (M)^	^TALEN1/2^	^pBLG-puro^	^332^	^26 (7.83)^	^20 (6.02)^	^2^
	^TALEN1/2^	^pBLG-hLF-puro^	^344^	^24 (6.97)^	^20 (5.81)^	^3^
^TGAF2 (F)^	^TALEN1/2^	^pBLG-puro^	^335^	^24(7.16)^	^19 (5.67)^	^3^
	^TALEN1/2^	^pBLG-hLF-puro^	^341^	^25 (7.33)^	^21 (6.16)^	^3^

^*^Junctions of the PCR-positive clones were scored as senescent when the cell numbers did not increase after 7 days.

**Table 2:** Germline transmission of *BLG*-targeted modification.

**F**_**0**_**(♂)**	**Female F**_**0**_**(♀)**	**Genotypes of F**_**1**_		
		**Wild type**	**BLG**^**+/−**^	**BLG**^**−/−**^
#02 (*BLG*^*+/−*^)	#05 (*BLG*^*+/−*^)	1	0	0
	#04 (*BLG*^*+/−*^)	0	0	1
	#06 (*BLG*^*+/−*^)	1	1	0
	#07 (*BLG*^*+/−*^)	0	1	0
	Wild type	3	1	0
