# Supplementary material for: Gene targeting by TALEN-induced homologous recombination in goats directs production of β-lactoglobulin-free, high-human lactoferrin milk
Source: Sci Rep. 2015 May 21;5:10482. doi: 10.1038/srep10482 (PMC5386245; doi:10.1038/srep10482)
Supplement: Supplementary Information [file srep10482-s1.doc]

Supplementary Information for

**Gene targeting by TALEN-induced homologous recombination in goats directs production of β-lactoglobulin-free, high-human lactoferrin milk**

**Chenchen Cui ^a,b,1^, Yujie Song ^a,b,1^ , Jun Liu ^a,b,1^, Hengtao Ge** **^a,b^, Qian Li ^a,b^ , Hui Huang ^a,b^, Linyong Hu ^a,b^, Hongmei Zhu ^a,b^ , Yaping Jin ^a,b,2^ and Yong Zhang ^a,b,2^**

^a^College of Veterinary Medicine, Northwest A&F University, Yangling 712100, Shaanxi, China.

^b^Key Laboratory of Animal Biotechnology, Ministry of Agriculture, Northwest A&F University, Yangling 712100, Shaanxi, China.

^1^These authors contributed equally to this work.

^2^ To whom correspondence should be addressed. E-mail: Y.Z. ([zhy1956@263.net](mailto:zhy1956@263.net)) or Y.J. (yapinjin@163.com)

*Corresponding author:* **Yong Zhang** Address: College of Veterinary Medicine, Northwest A&F University, Yangling 712100, Shaanxi, China. Telephone number: (+86) 029-87080085. E-mail: [zhy1956@263.net](mailto:zhy1956@263.net)

**Yaping Jin** Address: College of Veterinary Medicine, Northwest A&F University, Yangling 712100, Shaanxi, China. Telephone number: (+86) 029-87091802. E-mail: yapinjin@163.com

ZFN2

E1

ZFN3

ATCCAGGCCATCATCGTcacccaGACCATGAAAGGCCTG

TAGGTCCGGTAGTAGCAgtgggtCTGGTACTTTCCGGAC

ZFN4

GGCCTGGACATCCAGaaggtTCGAGGGTGGCCGGTGG

CCGGACCTGTAGGTCttccaAGCTCCCACCGGCCACC

CATGAAAGGCCTGGACATCcagaaGGTTCGAGGGTGGCC

GTACTTTCCGGACCTGTAGgtcttCCAAGCTCCCACCGG

E2

E3

E7

ZFN1

ZFN6

ZFN5

A

TALEN1

TALEN2

TCCAGGCCATCATCGTcacccagaccatgaaAGGCCTGGACATCCAGA

AGGTCCGGTAGTAGCAgtgggtctggtacttTCCGGACCTGTAGGTCT

TGCAACTCAAGGTCCCTCTccaggtggcggggacttggtACTCCTTGGCTATGGCGGCCA

ACGTTGAGTTCCAGGGAGAggtccaccgcccctgaaccaTGAGGAACCGATACCGCCGGT

TALEN3

TALEN4

ZFN activity for goat Beta Lactoglobulin

ZFN pairs

MEL-1 activity

B

**Figure S1. ZFNs and TALENs design against the goat *BLG* gene.** (A) Schematic of the goat *BLG* gene. The binding sites of ZFNs and TALENs are indicated by lines. (B) ZFN activity for goat *BLG* gene as measured by the yeast MEL-1 reporter assay. ZFN cleavage activity was measured before (0 h, blue bars) and after (6 h, red bars) induction of ZFN expression. MEL-1 levels were positively correlated with the ZFN ability to create double strand breaks at the desired target site. ZFNs with >50% signals relative to the positive control ZFN after induction (6 h) were considered useful for genome editing experiments.

A


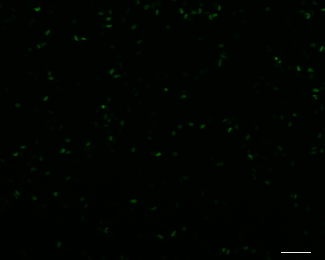

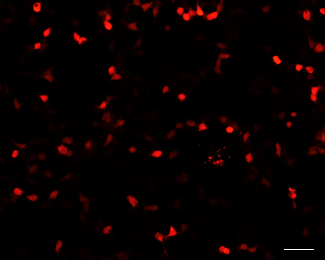

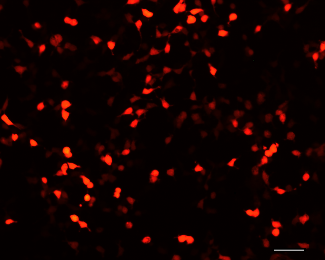

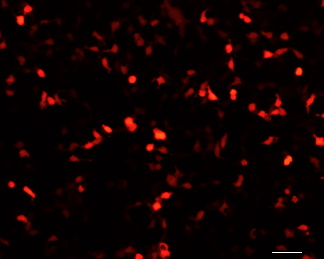

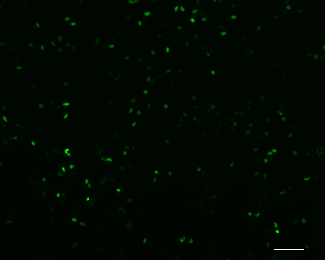

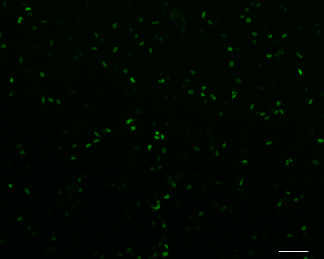


ZFN1/2 + Reporter

ZFN3/4 + Reporter

ZFN5/6 + Reporter


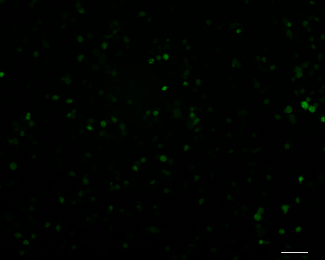

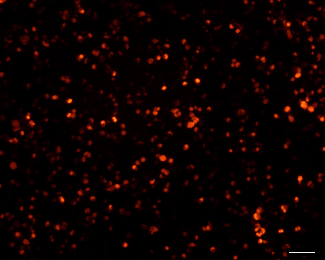

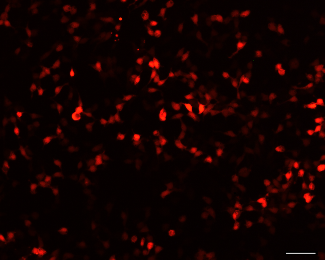

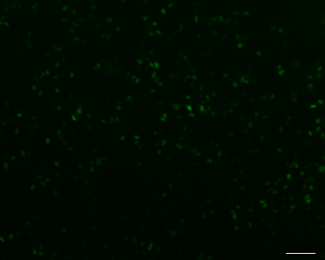

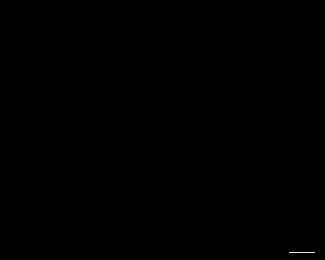

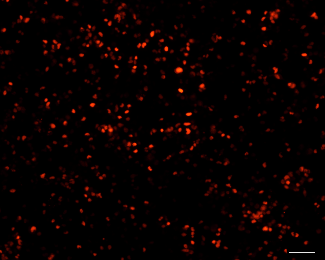


TALEN1/2 + Reporter

TALEN3/4 + Reporter

Reporter only

B

Repair by NHEJ

TALEN-induced DSB

pA

RFPrfpRF
P

GFP

CMV

ZFNs/TALENs target site

pA

RFP

GFP

CMV

pA

RFP

GFP

CMV

GFP

GFP

GFP

RFP

RFP

RFP

**Figure S2. Validation of ZFN and TALEN activity in 293FT cells.** (A) Schematic overview of the reporter system. CMV, human cytomegalovirus immediate early promoter; RFP, red fluorescent protein gene; GFP, green fluorescent protein gene; pA, polyadenylation signals. The *GFP* gene cloned into the reporter vector was a frameshift fragment. The wrong ORF can be repaired by NHEJ when TALENs cleaved the target site and induced NHEJ. (B) 293FT cells co-transfected with ZFNs/TALENs and their corresponding reporters. The scale bar indicates 50 μm.

## Table S1

Gene targeting in goat fibroblasts using TALEN-encoding mRNAs

| Cells lines  (Sex) | Targeting vector | Clones picked | PCR^+^ clones^*^ | senesced^**^ | Targeting efficiency (%) |
| --- | --- | --- | --- | --- | --- |
| GFF4 (F) | pBLG-neo-M | 236 | 25 | 5 | 10.6 |
|  | pBLG-hLF-neo | 267 | 25 | 7 | 9.36 |
| GFF2 (M) | pBLG-neo-M | 223 | 24 | 5 | 10.7 |
|  | pBLG-hLF-neo | 254 | 23 | 5 | 9.06 |
| TGAF1 (M) | pBLG-puro | 317 | 16 | 2 | 5.05 |
|  | pBLG-hLF-puro | 335 | 16 |  | 4.78 |
| TGAF2 (F) | pBLG-puro | 330 | 17 | 3 | 5.15 |
|  | pBLG-hLF-puro | 350 | 17 |  | 4.86 |

^*^ Clones were detected as *BLG*-targeted by two round of PCR analysis.

^**^PCR^+^ clones were scored as senesced when cell numbers could not be seen to increase after seven days.

Table S2

In vitro development of cloned embryos from different cell clones.

| Donor cell lines | Fusion rate^*^ | Cleavage rate^**^ | Blastocyst rate^***^ |
| --- | --- | --- | --- |
| 0622E4^†^ | (105/122)86.0±0.8 ^a^ | (77)73.4±0.9 ^ab^ | (22)20.8±1.4 ^a^ |
| 0622E36^†^ | (106/124)85.5±1.4 ^a^ | (77)72.7±1.4 ^ab^ | (22)20.7±0.3 ^ab^ |
| 0622E64^†^ | (103/122)84.4±2.2 ^a^ | (66)64.1±1.6 ^b^ | (17)16.6±1.9 ^b^ |
| 0919E08^‡^ | (109/128)85.2±1.3 ^a^ | (72)66.1±3.2 ^b^ | (19)17.4±0.9 ^b^ |
| 0919E39^‡^ | (105/125)84.0±3.6 ^a^ | (70)66.9±4.4 ^b^ | (18)17.1±1.6 ^b^ |
| 0919E22^‡^  0622 | (107/125)85.6 ±2.0 ^a^ | (78)72.9±3.0 ^ab^ | (17)15.9±1.8 ^b^ |
| 0919E111^‡^ | (109/125)87.2±1.2 ^a^ | (82)75.5±2.0 ^ab^ | (23)21.1±0.5 ^a^ |
| 0812E18^‡^ | (111/129)86.1±1.9 ^a^ | (81)73.1±0.4^a^ | (23)20.7±1.1 ^ab^ |
| 0812E62^‡^ | (110/128)84.0±0.6 ^a^ | (79)71.7±3.5 ^b^ | (20)17.3±1.1 ^b^ |
| GFF2 | (115/131)87.8±3.1 ^a^ | (88)76.5±2.7 ^a^ | (26)22.6±0.8 ^a^ |

^*^fusion rate = No. of fused embryos / No. of couplets.

^**^ cleavage rate = No. of cleavage embryos / No. of fused embryos.

^***^ blastocyst rate = No. of blastocyst / No. of fused embryos.

^a–c^ within a column, means without a common superscript differed (*P* < 0.05)

^†^cell clones derived from GFF2

^‡^cell clones derived from GFF4

Each cell clones was repeated four times.

Table S3

Summary of BLG-targeted goats

| Donor cells (sex) | Founders | Gestation length (day) | Birth weight  (kg) |
| --- | --- | --- | --- |
| 0622E4 (M) | #01 | 154 | 3.3 |
| 0622E36(M) | #02 | 150 | 2.9 |
|  | #03 | 150 | 3.1 |
| 0812E18(F) | #04 | 151 | 3.2 |
|  | #05 | 152 | 3.6 |
|  | #06 | 151 | 3.4 |
|  | #07 | 153 | 2.4 |
| LF06 (F) | #L1 | 154 | 3.3 |
|  | #L2 | 151 | 2.9 |
| LF24 (F) | #L3 | 152 | 3.0 |
| BT23(M) | #B1 | 153 | 2.8 |
| BT153(F) | #B2 | 154 | 3.5 |
|  | #B3 | 156 | 2.5 |
| LBT29 | #LB1 | 160 | 4.1 |
|  | #LB2 | 154 | 3.9 |
